# The dental complications of canine tooth bud removal in 2–12 years old children in Northwest Ethiopia

**DOI:** 10.1186/s13104-019-4743-9

**Published:** 2019-10-28

**Authors:** Amare Teshome, Berihun Assefa

**Affiliations:** 1 Department of Dentistry, School of Medicine, College of Medicine and Health Science, University of Gondar, P.o.box 196, Gondar, Ethiopia; 2 Department of Biostatics and Epidemiology, Institute of Public Health, College of Medicine and Health Science, University of Gondar, Gondar, Ethiopia

**Keywords:** Canine tooth bud removal, Infantile oral mutilation, Prevalence, Malformed canine, Missed canine

## Abstract

**Objective:**

Canine tooth bud removal is a process of gouging out an infant’s canine tooth buds, using unsterile tools such as Sharpe blade, garlic, or knitting needle, without anesthesia. The aim of the study was to reveal dental complications of canine tooth bud removal among children who visited the dental clinic of the University of Gondar hospital. This study was an institution-based cross-sectional conducted from January 2015 to September 2016 at the University of Gondar hospital on 2–12 years children. The tooth was assessed for whether it had previously oral mutilated or not. In addition to this, the oral cavity was evaluated for the presence of missed, malformed or normal canine.

**Results:**

A group of 355 children aged 2–12 years was examined clinically. The mean age of the children was 7.32 ± 3.12 (SD). The prevalence of canine tooth bud removal was 86.8% which was high in 6–9 years old (54.87%) and first position children (40.26%). The most common dental complications were; malformed enamel (hypoplastic) canine (48.5%) and missed/unerupted canine (38.6%).

## Introduction

Infant oral mutilation (IOM) is a traditional practice where a non-formally trained person gouges out un-erupted teeth, usually in the position of canines. The incidence of IOM is still rampant in the East African region and has been associated with cultural, geographic, ritual and aesthetic grounds [1–5]. Several terms are used to refer IOM that included tooth extirpation, canine tooth bud removal (CTBR), ebinyo, killer canine, nylon teeth and false teeth [6–9]. In Ethiopia, CTBR and the killer canine were commonly used in the literature [10–12].

Canine tooth bud removal (CTBR) is a process of gouging out an infant’s canine tooth buds using crude, an unsterile sharp tool such as; hooks, knives, knitting needle, bicycle spoke, a hot nail, a penknife or rubbing of the swollen gum with garlic without anesthesia [13–15]. This practice is usually done when the child is within 4–18 months of age and the gingival growth around the canine area is considered to be worm and they decide to remove this worm [14, 15].

The practice of CTBR is common in developing countries, especially in East Africa. The prevalence was different in each country of the region; Sudan (70%) [16], Kenya (35%) [17], Uganda (16.1%) [18], Tanzania (60.3%) [19] and 70% in Ethiopia [20]. This practice has been existing for many years in Africa due to the strong belief of the community as a preventive or curative measure of diarrhea, vomiting, fever, weight loss, failure to suck and retarded growth in children with unknown causes [1, 11, 16, 19, 21–25].

Community elders or traditional healers do the practice of CTBR. This practice was done with the assumption that gum swelling around the canine region is the cause of the childhood illness and removal of this canine, would avoid or cure these childhood diseases and prevent child death [13–15].

Across-sectional study done in pre-school children in Sudan found that Enamel hypoplasia was the most common dental anomaly (58.23%) and followed by localized enamel opacities (29%) in children with a previous history of IOM. There was also 12.6% of the complete absence of the mutilated tooth in the study participants. Mandibular canine (73.4%) was the most commonly affected tooth by this malpractice [26].

In order to develop appropriate and effective preventive measures towards the IOM practice, the extent of the problem and its effect on the developing dentition should be investigated. Therefore, this study is aimed at assessing the impact of CTBR in the dentition status.

## Main text

### Study design and study area

An institution-based descriptive cross-sectional study was conducted from January 2015 to September 2016 at the University of Gondar hospital. Gondar town is located 738 km Northwest of Addis Ababa, the capital of Ethiopia.

The study populations were children aged 2–12 years who visited the dental clinic of the University of Gondar hospital in the study period. The children not included in the study were, under 2 and above 12 years, those who failed to provide the consent, acutely sick on the day of the examination.

During data collection, all children (2–12 years) visited the University of Gondar hospital dental clinic were considered to participate in the study.

The sample size of the study was done using a single population

$${\text{n}} = \frac{{{\text{Z}}^{ 2} {\text{P}}\left( { 1- {\text{P}}} \right)}}{{{\text{d}}^{ 2} }},$$

where n = sample size, Z = Z statistic for a level of confidence (1.96), P = expected prevalence or proportion (a prevalence of 70% in the area according to the 1990 study [20]). d = precision (5%)

$${\text{n}} = \frac{{1.96^{2} \times 0.7(1 - 0.7)}}{{\left( {0.05} \right)^{2} }} = 322.$$

With 10% non-response rate (32) the total sample size was estimated to be 355.

A systematic sampling technique (every other child) was used in the data collection process.

### Data collection

Data collection was done using both questionnaires and oral examination

A structured closed-ended questionnaire was prepared to collect socio-demographic data and CTBR practices. Socio-demographic information for the children and parents was taken by face-to-face interviews of the parents/legal guardians.

The study had two parts, a face-to-face interview with the parents or guardians using structured closed-ended questionnaires to collect data on age, gender, child position, maternal educational status, maternal occupation, predisposing factors to IOM, and CTBR practice.

The second part of the data collection tool was oral examination to assess the status of the participant’s teeth.

### Face-to-face interview

A structured closed-ended questionnaire was used to collect data on age, gender, child position, maternal educational status, maternal occupation, predisposing factors to IOM, and CTBR practice. The questionnaire was initially piloted and tested by the two data collectors concerning the understandability of the questionnaire by the community before being used.

To assure the validity of the material a pilot study was done in 30 attendants before the actual data collection period to know the participants understand the questionnaire. The questionnaire was first written in English and translated to the local language (Amharic) and back to English again to check the validity of the language. A 2 days training was given for data collectors to brief them about the data collection instruments and the overall aim of the study and they collected the data using the pre-designed questioners under the strict supervision of the principal investigator.

### Oral examinations

The principal investigator at the dental OPD did oral examination of the included children using a disposable glove, portable torch, wooden spatula, mouth mirror, dental probes, and dental x-ray. A record on the number of teeth present in the jaw was made using the individual form. The tooth was assessed for whether it had previously oral mutilated or not. In addition to this, the oral cavity was evaluated for the presence of missing, malformed, or normal dentition. Missed is recorded when a child had a previous history of IOM and the tooth does not erupt within the expected time of the eruption and dental X-ray was used in some children. It was assumed malformed due to IOM if there was a history of tooth bud removal and the presence of enamel defect after the eruption.

### Ethical clearance and consent/assent

Ethical clearance was obtained from the institutional review board of the University of Gondar. The parents or guardians of the children were briefed for the aim of the study and asked for consent and informed voluntary assent was obtained without pressure from parents. Before they are allowed to participate in the study, they are told the participation is voluntary and no risks. They are allowed to take part in the study after signing the consent form. The parents or guardians were given oral health education on the impact of IOM practice on the general health of the child and tooth development.

### Data analysis

After coding and editing, data were entered and analyzed using SPSS version 20.0. Descriptive data were given in frequency and percentages depending on the variable type. Prevalence estimations were made for different Socio-demographic characteristics.

## Results

In this study, 355 children within 2–12 years old were evaluated and their parents/guardians were interviewed with a response rate of 100%. The number of male children was (51.83%) and the mean age of children was 7.32 ± 3.12. Majority of the respondents (84.8%) were orthodox Christians and within 6–9 (54.08%) years old (see Additional file 1).

The parents/guardians revealed that Diarrhea (65.26%) and Fever (15.26%) were the major morbidities that do occur during teething (Table 1). The prevalence of CTBR was 86.8% with a high prevalence within 6–9 (54.87%) years old, first position child (40.26%), orthodox (87.99%), and among a child with his/her mother’s occupation is a housewife (46.11) (see Additional file 2). There was a significantly high prevalence of CTBR in 5–9 years children (p = 0.002559) and those with a monthly income of < 1000 birr (p = 0.016517) (see Additional file 2).

**Table 1:** The major symptoms that cause canine tooth bud removal in the study participants (n = 308), 2015/2016

Symptoms	Frequency	Percentage
Diarrhea	201	65.26
Vomiting	24	7.79
Fever	47	15.26
Body wasting	36	11.69

The missed and malformed canine was the major dental complications observed in oral mutilated children. Only 12.9% of children with a history of IOM had normal canine (Table 2).

**Table 2:** Deciduous canine status of children visiting dental clinic of University of Gondar hospital (n = 308), 2015/2016

Canine status	Frequency	Percent
Intact/normal	40	12.9
Malformed enamel	149	48.5
Missing	119	38.6
Total	308	100

**Fig. 1:**
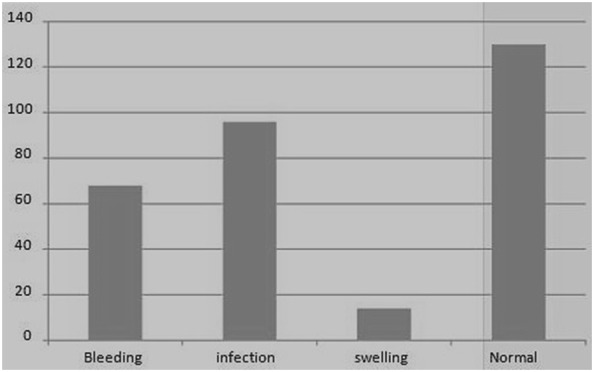
Post extraction complications among children visiting dental clinic of university of Gondar hospital (n = 308), 2015

Out of three hundred eight children, CTBR, one hundred seventy-eights had post-extraction complications. The common complications were bleeding and infection (Fig. 1).

## Discussion

The result of the present study revealed that the practice of CTBR was 86.8% which is similar to the study done in Northwest Ethiopia (84.5%) in 1990 [20]. However, this finding is higher when compared with the studies done in Sudan (70%) [16], Kenya in 1988 (35%) [17] and 1995 (72%) [22],Uganda (16.1) [18], Tanzania (60.3%) [19], and Ethiopia (59%) [11]. This is due to the deep-rooted beliefs, customs and attitudes, and lack of knowledge and awareness on the long and short complications of the practice in the study area [23].

The practice of CTBR is high within 6–9 (54.87%) years old (p = 0.002559) and first position (40.26%) children. This result showed the practice is decreasing in a newborn child, maybe due to the expansion of health extension programs and now the awareness of the parents is growing.

In this study, nearly 90% of children with a history of oral mutilated, presented with either malformed or missed canines. These defects result from the failure of the complete removal of the tooth bud. According to this study, the prevalence of malformed canine was 48.5% followed by missed (38.6%) canine. This is low when compared with the study done in the Maasai community in Kenya [22], where 72% of the children had missed canine. However, this result is high when compared with the study done in the offspring of Ethiopians in Israel, where there was 29.6–31.1% of malformed enamel and 26.6–29.8% of them had missed canine [27]. This might be due to the variation in culture, accessibility, and affordability of dental facilities in these two countries and there is a strong belief in traditional healers and lack of awareness on the negative effects of the practice in the study area.

Only three patients (0.85%) had maxillary canine removed, which is comparable with the study done in the offspring of Ethiopian immigrants in Israel [27], where only one upper canine was removed. The majority (69.89%) of the participants had bilateral mandibular canine mutilated, of which, 49.46% of them had missed canine in both sides. This finding is low compared with the finding in the offspring of Ethiopian immigrants (84.4%) of missed canine in both sides [27]. This might be due to incomplete removal of the canine tooth bud during IOM due to some change in the use of materials during oral mutilation (like garlic rub) in the study area.

Infections (53.63%) and bleeding (37.99%) were the major problems facing the child after the procedure was done. The parents don’t report the presence of blood born infection. This finding supports previous studies where the use of unsterile Sharpe material increases the risk of bleeding, infection, and damage of the permanent tooth bud [17, 19, 22–24].

## Conclusion and recommendation

The practice of CTBR/IOM affects the most delicate structure of the developing tooth. Canine malformation and missing were the two major complications. There must be a multi-disciplinary approach to combat the occurrence of this harmful practice.

## Limitations

The authors faced the following limitations during conducting this research.

- Some of the parents are not willing to tell their child CTBR practice.
- The present study sample represents the population living in Gondar town and around areas and cannot be extrapolated to Ethiopia in general. So, large-scale longitudinal studies are needed.

## Supplementary information

**Additional file 1.** Sociodemographic characteristics of children visited the Dental Clinic of Gondar University Hospital (n = 355), 2015/16.

**Additional file 2.** The CTBR practice among children visited the Dental clinic of the University of Gondar Hospital, 2015/16.

## Data Availability

All the data obtained during this study are available within the article.

## Abbreviations

CTBR: canine tooth bud removal
IOM: infant oral mutilation
OPD: outpatient department
