# Supplementary material for: The dental complications of canine tooth bud removal in 2–12 years old children in Northwest Ethiopia
Source: BMC Res Notes. 2019 Oct 28;12:701. doi: 10.1186/s13104-019-4743-9 (PMC6816202; doi:10.1186/s13104-019-4743-9)
Supplement: Supplementary file 1 — Additional file 1. Sociodemographic characteristics of children visited the Dental Clinic of Gondar University Hospital (n = 355), 2015/16. [file 13104_2019_4743_MOESM1_ESM.docx]

**File 1:**  Sociodemographic characteristics of children visited Dental Clinic of Gondar University Hospital (n=355), 2015/16.

|  | **Sociodemographic Characteristics** | | | |  | Frequency | |  | Percentage | |
| --- | --- | --- | --- | --- | --- | --- | --- | --- | --- | --- |
|  |  | |  |  |  |  |  |  |  |  |
|  | **Gender of child** | |  | Male |  | 184 |  |  | 51.83 |  |
|  |  |  |  | Female |  | 171 |  |  | 48.17 |  |
|  |  |  |  |  |  |  |  |  |  |  |
|  |  | |  |  |  |  |  |  |  |  |
|  | **Age of child (years)** | | 2-5 | | 73 | |  | 20.56 | |  |
|  |  |  | 6-9 | | 192 | |  | 54.08 | |  |
|  |  |  | 10-12 | | 90 | |  | 25.36 | |  |
|  |  | |  |  |  | |  |  | |  |
|  | **Position of child** | |  | First |  | 145 |  |  | 40.85 |  |
|  |  |  |  |  |  |  |  |  |  |  |
|  |  |  |  | Second |  | 108 |  |  | 30.42 |  |
|  |  |  |  | Third |  | 61 |  |  | 17.18 |  |
|  |  |  |  | Fourth |  | 33 |  |  | 9.29 |  |
|  |  |  |  | Above fourth |  | 8 |  |  | 2.26 |  |
|  |  |  |  |  |  |  |  |  |  |  |
|  |  |  |  |  |  |  |  |  |  |  |
|  | **Religion** |  |  | Orthodox | 311 | |  | 87.61 | |  |
|  |  |  |  | Muslim | 28 | |  | 7.89 | |  |
|  |  |  |  | Protestant | 16 | |  | 4.50 | |  |
|  |  |  |  |  |  | |  |  | |  |
|  | **Maternal** | **educational** |  | <grade 8 |  | 159 |  |  | 44.79 |  |
|  | **level** |  |  | ≥grade 8 |  | 196 |  |  | 55.21 |  |
|  |  | |  |  |  |  |  |  |  |  |
|  | **Occupation of mothers** | |  | Housewife | 163 | |  | 45.92 | |  |
|  |  |  |  | Businesswomen | 72 | |  | 20.29 | |  |
|  |  |  |  | Farmer | 8 | |  | 2.25 | |  |
|  |  |  |  | Governmental | 104 | |  | 29.29 | |  |
|  |  |  |  | employee |  |  |  |  |  |  |
|  |  | |  |  |  | |  |  | |  |
|  | **Monthly family income*** | |  | <1000 Birr |  | 123 |  |  | 34.65 |  |
|  |  |  |  | ≥1000 Birr |  | 232 |  |  | 65.35 |  |
|  |  |  |  |  |  |  |  |  |  |  |
|  |  |  |  |  |  |  |  |  |  |  |

* Ethiopian birr
