# Supplementary material for: The dental complications of canine tooth bud removal in 2–12 years old children in Northwest Ethiopia
Source: BMC Res Notes. 2019 Oct 28;12:701. doi: 10.1186/s13104-019-4743-9 (PMC6816202; doi:10.1186/s13104-019-4743-9)
Supplement: Supplementary file 2 — Additional file 2. The CTBR practice among children visited the Dental clinic of the University of Gondar Hospital, 2015/16. [file 13104_2019_4743_MOESM2_ESM.docx]

**File 2:** The oral mutilation practice among children visited Dental clinic of University of Gondar Hospital, 2015/16.

| Sociodemographic characteristic | | Infantile oral mutilation is done | | P value/Chi square |
| --- | --- | --- | --- | --- |
| Gender of the child | Male | Yes | No |  |
|  |  | 157 | 27 | X ^2:^ 0.6843.  *p*-value 0.408111 |
|  | Female | 151 | 20 |  |
| Age of the child | 2-5 years | 55 | 18 | X^2:^  11.9359.  *p*: 0.002559 |
|  | 6-9 years | 169 | 23 |  |
|  | 10-12 years | 84 | 6 |  |
| Birth order of the child | Firstborn | 124 | 21 | ^X2 :^ 4.5494.  *p*-value : 0 .336733 |
|  | Secondborn | 95 | 13 |  |
|  | Third | 50 | 11 |  |
|  | Fourth | 31 | 2 |  |
|  | Fifth and above | 7 | 1 |  |
| Religion | Orthodox | 271 | 40 | X^2^ : 2.1206.  P value: 0.346348 |
|  | Muslim | 25 | 3 |  |
|  | Protestant | 12 | 4 |  |
| Maternal educational level | <grade 8 | 139 | 20 | X^2^ : 0.1095.  P value: 0.740734 |
|  | >Grade 8 | 169 | 27 |  |
| Occupation of the mother | Housewife | 142 | 21 | X^2^: 12.5662  *p*-value: 0.013602 |
|  | Private business | 55 | 15 |  |
|  | Farmer | 6 | 2 |  |
|  | Governmental employee | 98 | 6 |  |
|  | NGO employee | 6 | 2 |  |
| Familly monthly income (birr) | <1000 birr | 114 | 9 | X^2^: 5.747  p-value: 0.016517 |
|  | >1000 birr | 194 | 38 |  |
